# Eco-Physiological Traits Related to Recovery from Complete Submergence in the Model Legume *Lotus japonicus*

**DOI:** 10.3390/plants9040538

**Published:** 2020-04-21

**Authors:** Florencia B. Buraschi, Federico P.O. Mollard, Agustín A. Grimoldi, Gustavo G. Striker

**Affiliations:** 1 IFEVA, CONICET, Cátedra de Fisiología Vegetal, Facultad de Agronomía, Universidad de Buenos Aires, Av. San Martín 4453, Buenos Aires C1417DSE, Argentina; fburaschi@agro.uba.ar (F.B.B.); fmollard@agro.uba.ar (F.P.O.M.); 2 IFEVA, CONICET, Cátedra de Forrajicultura, Facultad de Agronomía, Universidad de Buenos Aires, Av. San Martín 4453, Buenos Aires C1417DSE, Argentina; grimoldi@agro.uba.ar; 3 UWA, School of Agriculture and Environment, Faculty of Science, The University of Western Australia, 35 Stirling Hwy, Crawley, WA 6009, Australia

**Keywords:** post-submergence recovery, legumes, plant water relations, shoot to root ratio, *Lotus japonicus*, leaf greenness, leaf desiccation, stomatal conductance

## Abstract

Submergence is a severe form of stress for most plants. *Lotus japonicus* is a model legume with potential use in assisting breeding programs of closely related forage *Lotus* species. Twelve *L.* *japonicus* genotypes (10 recombinant inbred lines (RILs) and 2 parental accessions) with different constitutive shoot to root dry mass ratios (S:R) were subjected to 7 days of submergence in clear water and allowed to recover for two weeks post-submergence; a set of non-submerged plants served as controls. Relative growth rate (RGR) was used to indicate the recovery ability of the plants. Leaf relative water content (RWC), stomatal conductance (g_s_), greenness of basal and apical leaves, and chlorophyll fluorescence (Fv/Fm, as a measure of photoinhibition) were monitored during recovery, and relationships among these variables and RGR were explored across genotypes. The main results showed (i) variation in recovery ability (RGR) from short-term complete submergence among genotypes, (ii) a trade-off between growth during vs. after the stress indicated by a negative correlation between RGR during submergence and RGR post-submergence, (iii) an inverse relationship between RGR during recovery and S:R upon de-submergence, (iv) positive relationships between RGR at early recovery and RWC and g_s_, which were negatively related to S:R, suggesting this parameter as a good estimator of plant water balance post-submergence, (v) chlorophyll retention allowed fast recovery as revealed by the positive relationship between greenness of basal and apical leaves and RGR during the first recovery week, and (vi) full repair of the submergence-damaged photosynthetic apparatus occurred more slowly (second recovery week) than full recovery of plant water relations. The inclusion of these traits contributing to submergence recovery in *L. japonicus* should be considered to speed up the breeding process of the closely related forage *Lotus* spp. used in current agriculture.

## 1. Introduction

As a result of the effects of climate change, an increase in the frequency and intensity of flooding events is expected to occur in the coming years [1]. A higher intensity of flooding can easily lead to plant submergence. Complete submergence denotes a condition where floodwaters increase to a level where plant shoots remain fully underwater; it is one of the most stressful scenarios that plants confront in prone-to-flood environments [2]. This situation drastically decreases the direct exchange of gases between the plant and the atmosphere, resulting in reduced O_2_ and CO_2_ levels [3]. Moreover, complete submergence often reduces the irradiance for photosynthesis depending on the water depth and turbidity. Two plant strategies have been recognized in plant submergence responses: (i) the “escape strategy” and (ii) the ”quiescent’ strategy” [4,5,6]. The first one involves shoot elongation to restore leaf contact with the atmosphere and offers plants a better chance to survive under shallow long-term submergence (>1 week). The ”quiescent” strategy is based on maintaining steady energy conservation without shoot elongation and it is usually adopted to cope with deep short-term submergence (<1 week), given that shoot emergence represents a high energy cost that could compromise subsequent plant recovery [3,7]. Thus, in scenarios of short, deep submergence with low CO_2_ and/or low light, plants rarely grow, but instead aim just to survive until the water recedes and later resume growth. Additionally, intraspecific variability between the escape and quiescent strategies has been found in rice [4]. The resumption of vigorous growth after experiencing complete submergence indicates the recovery ability of the species/genotype/accession [8]. Plant responses during submergence have been extensively studied and documented [4,9,10,11,12], while plant responses after water recession (i.e. recovery phase) have seldom been reported (but see [13,14] for *Arabidopsis thaliana*). This study aims at identifying eco-physiological traits facilitating plant recovery after complete submergence.

Forage legumes are important components of pastures and natural grasslands. The *Lotus* genus includes more than 180 species distributed worldwide [15,16], and it is used to improve pastures in stressful environments, where traditional forage legumes, such as lucerne or red clover, fail to grow or survive [17]. *Lotus corniculatus*, *L. pedunculatus*, and *L. tenuis* have been domesticated and improved through breeding for possessing some degree of tolerance to soil waterlogging, with *L. tenuis* being the most tolerant to root oxygen deficiency [6,18,19]. A few years ago, *L. japonicus*, which is taxonomically closely related to these forage species [20], arose as a model for legumes because it is a small plant with short generation time, easy cultivation, and it is amenable to transformation. So, a set of genetic resources was developed, including ecotypes, mutant lines, and recombinant inbred lines (RILs) [21,22]. Previous studies showed that adult plants of this species could tolerate three weeks of waterlogging [23] and up to 12 days of submergence at the seedling stage [6]. So, the characterization of *L. japonicus* responses to submergence and its post-submergence recovery can help support plans intended for the improvement of submergence tolerance of the closely related forage *Lotus* species.

Relevant traits for the recovery of plants from water submergence have scarcely been identified. It is perceived that plant water status is critical to facilitate fast recovery, particularly considering that a diminished and/or damaged root system due to submergence cannot necessarily cope with the water uptake needed to meet shoot transpiration when re-exposed to atmospheric air upon de-submergence ([24] for the grass *Chloris gayana*). In rice cv. IR42, the intolerance to complete submergence is caused by desiccation of leaves (i.e., decreased hydraulic conductivity in the leaf sheath) after de-submergence, which occurs rapidly, provoking wilting and death of the plants [25]. In this regard, shoot to root dry mass ratio upon de-submergence might be roughly related to water balance (i.e., transpiration by the shoot/water uptake by roots) and might influence plant recovery. Carbon assimilation after submergence can also positively impact plant recovery. Pioneer works [26,27] showed that the ability to resume growth after 20 days of submergence in the forb *Alternanthera philoxeroides* and the grass *Hemarthria altissima* resulted from rapid leaf growth and recovery of functionality of the photosynthetic apparatus. In two contrasting accessions for submergence tolerance of *Arabidopsis thaliana* (Bay-0-sensitive vs. Lp2-tolerant), it was shown that quick stomatal aperture, higher chlorophyll retention, and leaf water turgor maintenance were key factors for the tolerant accession to sustain a fast recovery following the removal of the stress [13], these traits also provide a detailed recovery-signaling network for enhanced flooding tolerance in *Arabidopsis*. In this study, we selected 10 RILs of *L. japonicus* (plus both parents) with differential constitutive shoot to root dry mass ratios (S:R) from [23] with the objective to assess leaf water status, stomatal conductance, dark-adapted chlorophyll fluorescence of photosystem II (Fv/Fm), and greenness of basal and apical leaves (as a surrogate to infer N status) and explore how these variables correlate with plant growth resumption (i.e., plant RGR: relative growth rate). We investigated these plant responses and relationships during the first and the second week after de-submergence to unveil traits that aid in early and late recovery. We hypothesized that there is variation among genotypes (i.e., RILs) of *Lotus japonicus* in their ability to recover from complete submergence associated with differences in the S:R ratio, plant water status, and leaf greenness. We expected that genotypes with low S:R present quick recovery of leaf water status, stomatal conductance, and high retention of leaf chlorophyll during the early recovery and, therefore, show high plant RGR post-submergence.

## 2. Results

### 2.1. Plant Growth during Submergence 

**Figure 1:**
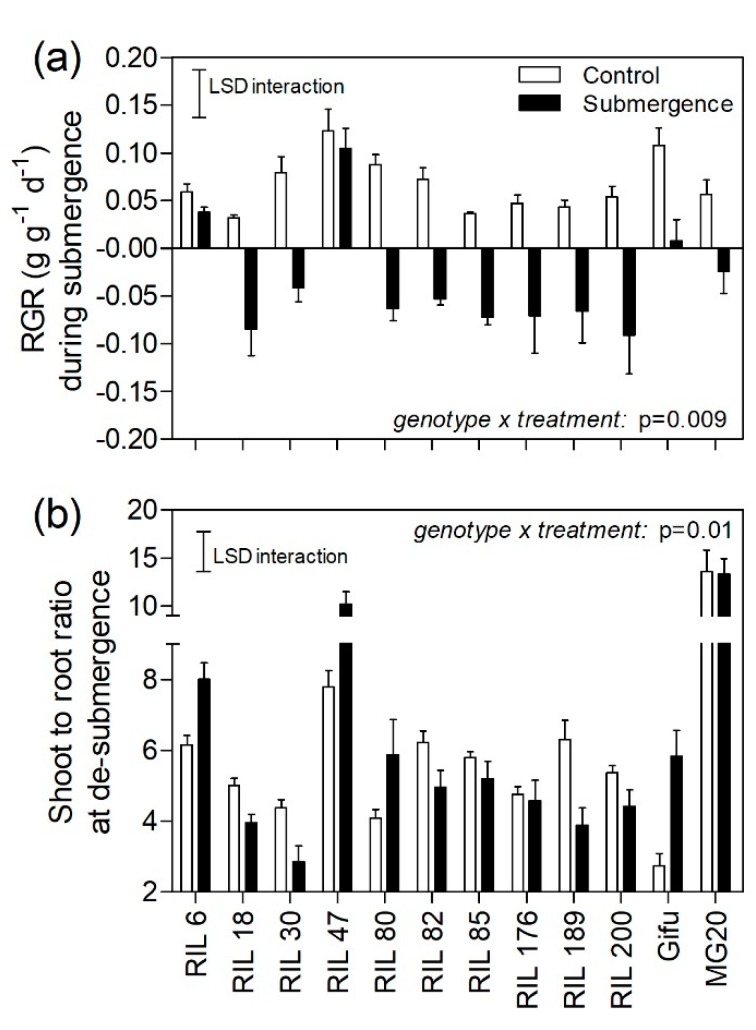
(**a**) Plant relative growth rate (RGR, g g-1 d-1) of 12 genotypes of *Lotus japonicus* (10 recombinant inbred lines and their parents Gifu and MG20) subjected to control and submergence in clear water for one week. (**b**) Shoot to root dry mass ratio (S:R) after the first week of the experiment both at control and de-submergence. The least significative difference (LSD) for the genotype × treatment interaction is shown in (a) (LSD RGR = 0.065) and (b) (LSD S:R = 3.7). Values are means ± standard errors of 6–8 replicates.

All genotypes (parents MG-20, Gifu, and 10 RILs) showed positive relative growth rates (RGR) during the first week of the experiment when under control conditions (Figure 1a). In contrast, 9 out of 12 genotypes (including the parent MG20) showed negative RGR during the one-week submergence, indicating the inability to accumulate biomass underwater and loss of tissues due to the stress (Figure 1a); Gifu and RILs 6 and 47 presented slight but positive values for RGR. In all cases/genotypes, plants were not able to emerge from the water, so the registered growth responses corresponded to plants that remained underwater for the whole week. Plant dry mass values from which RGR’s were calculated are available in Table S1.

Shoot to root dry mass ratio (S:R) of the selected RILs and their parents under control conditions ranged from 2.7 to 13.5 (with a median of 5; Figure 1b), which was wide enough to explore the relationship of this variable with plant recovery from submergence as one of our main objectives. Importantly, differences in S:R ratio among genotypes were constitutive and not related to plant size, as there was no significant relationships between plant dry mass and S:R (r^2^ = 0.34; *p* = 0.23). Four out of 12 genotypes, RILs 6, 47, 80, and Gifu, increased the S:R in response to complete submergence, while the other three genotypes, RILs 18, 30 and 189, decreased the S:R (Figure 1b). Interestingly, three out of the four genotypes that increased the S:R also showed positive RGR during submergence (RILs 6 and 47, and Gifu).

### 2.2. Plant Recovery from Submergence 

To assess plant recovery, we used plant relative growth rate after de-submergence as an indicator of the ability of the different genotypes to resume growth. RGR values for the first week of recovery were used to infer capacity for an ‘early recovery’ and RGR values of the second week to infer ‘late recovery’ responses. In this context, we analyzed how the ability to grow after submergence during these periods (i.e., RGR) correlated across genotypes with several parameters of interest such as (i) RGR during the submergence week per se, (ii) shoot to root dry mass ratio at de-submergence, (iii) leaf relative water content, (iv) stomatal conductance, (v) chlorophyll fluorescence of dark-adapted leaves (Fv/Fm), and (vi) greenness in basal, apical, and new leaves that appeared during the early and late weeks of recovery.

**Figure 2:**
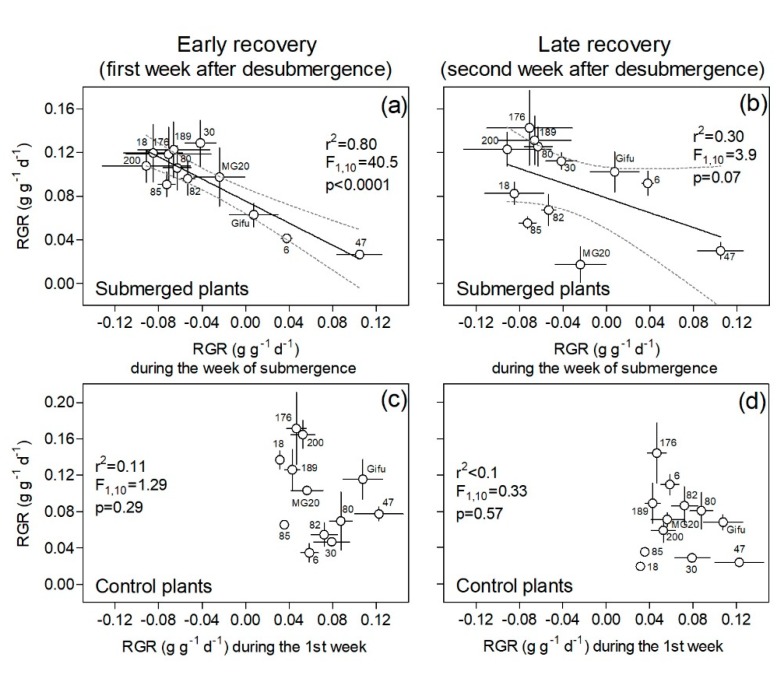
Plant relative growth rate (RGR) during ‘early’ and ‘late’ recovery vs. RGR during the first experimental week for one-week submergence (**a**,**b**) and control (**c**,**d**) treatments of 12 genotypes of *Lotus japonicus* (10 recombinant inbred lines and their parents Gifu and MG20). The first week after de-submergence was used to explore the relationship between these variables in an ‘early recovery’ phase (**a**,**c**) and the second week in a ‘late recovery’ phase (**b**, **d**). Values are means ± standard errors of 6–8 replicates.

RGR during the first week after submergence (i.e., early recovery) was inversely related to RGR during submergence across genotypes (Figure 2a). During the second week (i.e., late recovery), there was a trend (*p* = 0.07) towards maintenance of a negative relationship between the RGR during submergence and the RGR in the recovery (Figure 2b). So, in general, the genotypes that showed positive RGR during submergence were the poorest performers in terms of RGR during recovery phases (e.g., RILs 6 and 47, and Gifu). In the case of plants growing under control conditions, RGR during the first or second week of recovery (i.e., 2nd and 3rd weeks of the experiment, respectively) were not related to the RGR of these genotypes during the first week of the experiment (*p* > 0.29 in both cases; Figure 2c,d).

**Figure 3:**
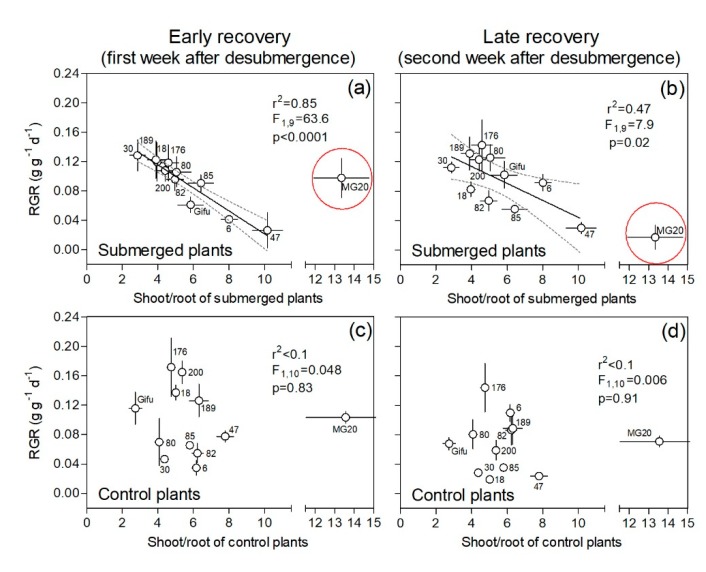
Plant relative growth rate (RGR) during ‘early’ and ‘late’ recovery vs. shoot to root dry mass ratio at the end of the first experimental week for one week of submergence (**a**,**b**) and control (**c**,**d**) treatments of 12 genotypes of *Lotus japonicus* (10 recombinant inbred lines and their parents Gifu and MG20). The first week after de-submergence was used to explore the relationship between these variables in an ‘early recovery’ phase (**a**,**c**) and the second week in a ‘late recovery’ phase (**b**,**d**). The MG20 genotype was considered an outlier and was not included in the regression parameters shown in (**a**) and (**b**); however, if included, parameters were r^2^ = 0.34, F_1,10_ = 5.33, and *p* = 0.043 and r^2^ = 0.63, F_1,10_ = 17.50, and *p* = 0.0019, respectively. Values are means ± standard errors of 6–8 replicates.

Shoot to root dry mass ratio (S:R) of submerged plants as an estimator for the potential of transpiration/water uptake under drained conditions showed a negative relationship with RGR during the first week of recovery across genotypes for plants coming from a week of submergence. This means that genotypes with low S:R (e.g., RILs 18, 30, 189) consistently displayed higher RGR during the early recovery (Figure 3a). Even though this negative relation between RGR and S:R persisted during the late recovery (2nd week after submergence), the fitting was weaker than during the early recovery (compare adjustment parameters in Figure 3a vs. Figure 3b). The genotype MG20 was not included in the regressions mentioned above as its S:R was extremely high and was therefore considered an outlier (i.e., S:R_MG20_ > S:R_average_ + 3 standard error). Under control conditions, there was no relation between RGR and S:R ratio either for the 2nd or the 3rd weeks of the experiment (*p* > 0.8 in both cases; Figure 3c,d).

### 2.3. Leaf Physiological Variables Related to Plant Recovery 

**Figure 4:**
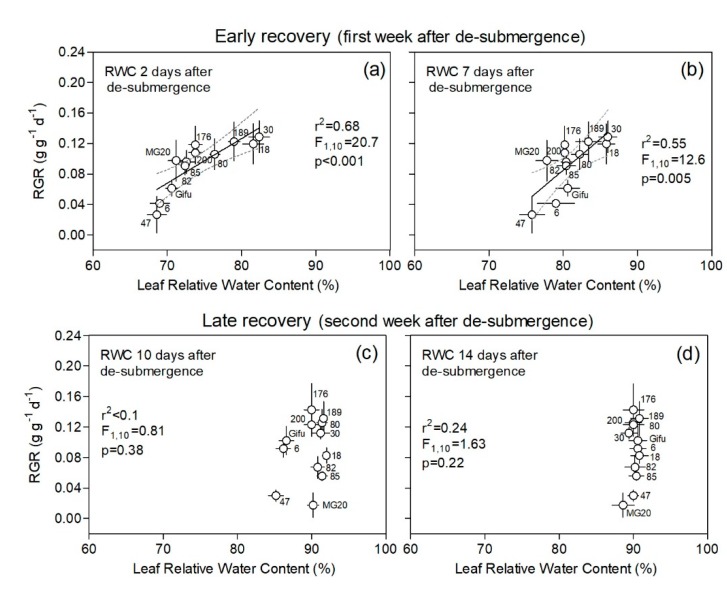
Plant relative growth rate (RGR) during ‘early’ and ‘late’ recovery vs. relative water content (RWC, %) on the top-most fully expanded leaves at days 2, 7, 10, and 14 after de-submergence for plants of 12 genotypes of *Lotus japonicus* (10 recombinant inbred lines and their parents Gifu and MG20). The first week after de-submergence was used to explore the relationship between these variables in an ‘early recovery’ phase (**a**,**b**) and the second week in a ‘late recovery’ phase (**c**,**d**). Values are means ± standard errors (n = 6–8 for RGR and n = 5 for leaf RWC). The leaf RWC values corresponding to the control treatment ranged between 89.2% and 92.4% (all data for each genotype and measurement dates are available in Table S2).

Leaf relative water content (leaf RWC), which reflects the balance between water supply to leaf tissue and transpiration rate, showed a positive relationship with RGR of plants upon de-submergence across genotypes during the first week of recovery (Figure 4). This positive relationship, where a higher RWC of the top-most fully expanded leaves corresponded to higher RGR, was true when correlating RGR of the first recovery week with leaf RWC at 2 and 7 days after de-submergence (Figure 4a,b). After ten days of de-submergence most genotypes fully recovered the values of leaf water status, except for RILs 6 and 47 and Gifu (Figure 4c). At the end of the second week of recovery (day 14), all genotypes showed high leaf RWC (Figure 4d), which resulted in similar values to those shown by control plants throughout the experiment (leaf RWC ranged from 89.2% to 92.1%; see Table S2).

**Figure 5:**
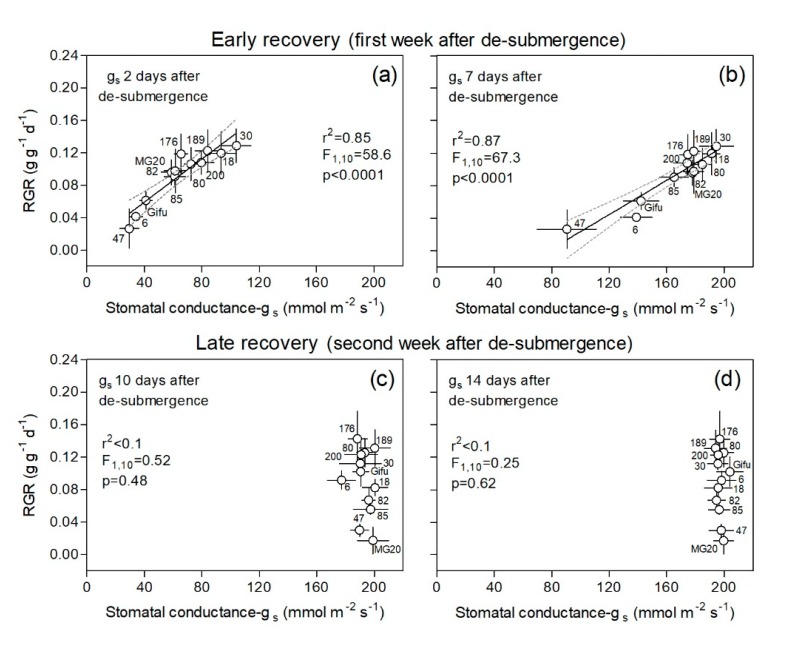
Plant relative growth rate (RGR) during ‘early’ and ‘late’ recovery vs. stomatal conductance (g_s_, mmol m^−2^ s^−1^) on the top-most fully expanded leaves at days 2, 7, 10, and 14 after de-submergence for plants of 12 genotypes of *Lotus japonicus* (10 recombinant inbred lines and their parents Gifu and MG20). The first week after de-submergence was used to explore the relationship between these variables in an ‘early recovery’ phase (**a**,**b**) and the second week in a ‘late recovery’ phase (**c**,**d**). Values are means ± standard errors (n = 6–8 for RGR and n = 5 for g_s_). The stomatal conductance values corresponding to the control treatment ranged between 187.2 and 208.8 mmol m^−2^ s^−1^ (all data for each genotype and measurement dates are available in Table S3).

Stomatal conductance (g_s_) estimates the rate of gas exchange (uptake of CO_2_ favoring photosynthesis) and transpiration (loss of water) through the leaf stomata as determined by the degree of stomatal aperture, which is in turn related to the leaf water status. For this reason, a similar response pattern of g_s_ and leaf RWC was not unexpected (Figure 5). In this regard, we found a positive relationship between the RGR of plants upon de-submergence across genotypes and g_s_ on the top-most fully expanded leaves during the first week of recovery (Figure 5). This relationship, where higher g_s_ values paralleled higher RGRs, was significant when correlating RGR of the first recovery-week with g_s_ values at 2 and 7 days after de-submergence (Figure 4a,b). Nevertheless, it should be noticed that after reaching one week after de-submergence (day 7), values of g_s_ were increasing and were close to full recovery (compared to controls; see Table S3), with the exception of genotypes RILs 6 and 47 and Gifu, which remained with the lowest values for this parameter (Figure 5b). At 10 and 14 days after de-submergence (second recovery week), all genotypes presented similar values for g_s_ as those of the controls for all genotypes (Figure 4c,d). Measured values in control plants of these genotypes through the experiment ranged between 187 and 208 mmoles m^−2^ s^−1^ (Table S3).

**Figure 6:**
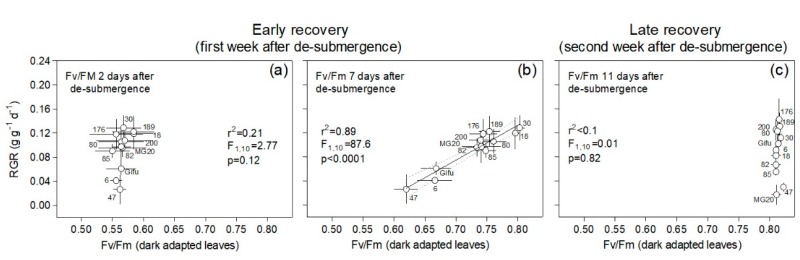
Plant relative growth rate (RGR) during ‘early’ and ‘late’ recovery vs. chlorophyll fluorescence on the top-most fully expanded dark-adapted leaves (Fv/Fm) at days 2, 7, and 11 after de-submergence for plants of 12 genotypes of *Lotus japonicus* (10 recombinant inbred lines and their parents Gifu and MG20). The first week after de-submergence was used to explore the relationship between these variables in an ‘early recovery’ phase (**a**,**b**) and the second week in a ‘late recovery’ phase (**c**). Values are means ± standard errors (n = 6–8 for RGR and n = 5 for Fv/Fm). The Fv/Fm values corresponding to the control treatment ranged between 0.803 and 0.816 (all data for each genotype and measurement dates are available in Table S4).

A decrease in chlorophyll fluorescence of dark-adapted leaves (Fv/Fm) below 0.8 is considered as an indicator of photoinhibition, particularly related to damage to Photosystem II. Interestingly, plants of all genotypes showed low (and similar) values for Fv/Fm (from 0.55 to 0.59) on the top-most fully expanded leaves when measured two days after de-submergence, and no relation with plant RGR during the first recovery week was found across genotypes (Figure 6a). After one week of recovery, Fv/Fm was positively correlated to RGR across genotypes with the poorest performers, namely RILs 6 and 47 and Gifu, still presenting low values indicative of photodamage. During the second recovery week, the possible measurement was made at 11 days after de-submergence, and values for all genotypes showed full recovery for this parameter without differences with their corresponding controls (Table S4). In the case of control plants, the registered values through the experiment ranged between 0.803 and 0.816, considering all genotypes (Table S4).

**Figure 7:**
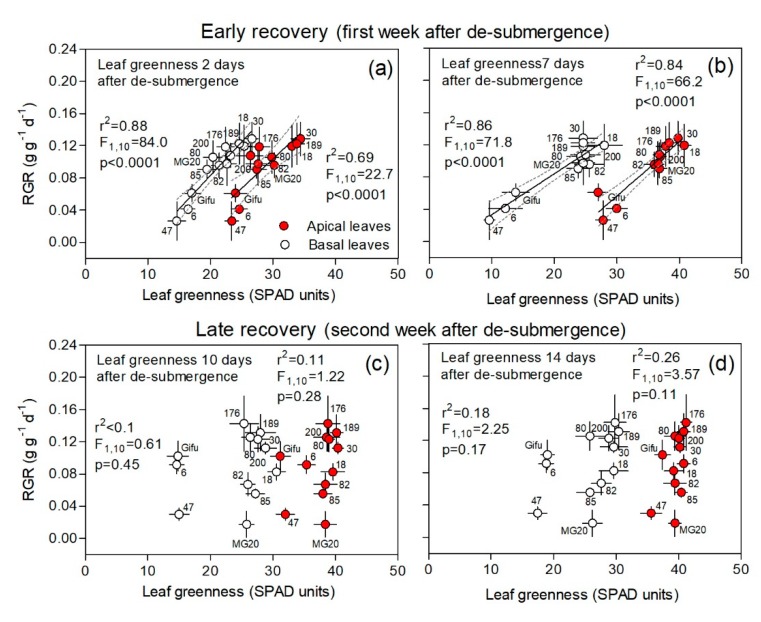
Plant relative growth rate (RGR) during ‘early’ and ‘late’ recovery vs. greenness (SPAD units) of basal and apical fully expanded leaves at days 2, 7, 10, and 14 after de-submergence for plants of 12 genotypes of *Lotus japonicus* (10 recombinant inbred lines and their parents Gifu and MG20). The first week after de-submergence was used to explore the relationship between these variables in an ‘early recovery’ phase (**a**,**b**) and the second week in a ‘late recovery’ phase (**c**,**d**). Values are means ± standard errors (n = 6–8 for RGR and n = 5 for greenness). The leaf greenness values corresponding to the control treatment ranged between 33.6 and 37.6 SPAD units for basal leaves and 33.6 and 37.6 SPAD units for apical young fully expanded leaves (all data for each genotype and measurement dates are available in Table S5).

Leaf greenness monitoring throughout the experiment after de-submergence in basal and apical positions allows for the estimation of the nitrogen status of leaves, which is in general related to the potential for photosynthesis and, in the case of basal leaves, it allows us to infer senescence and nitrogen remobilization. We found that one week of submergence determined lower greenness values in both basal and apical leaves when compared to controls (see values in Figure 7a,b vs. Table S5). In turn, RGR during the first recovery week was positively related to greenness of both basal and apical leaves across genotypes, both at 2 and 7 days after de-submergence. This means that genotypes that retained more chlorophyll (RILS 18, 30, and 189) showed steadily higher growth (Figure 7a,b). During the second week of recovery, there was no clear relationship between plant RGR and leaf greenness. However, it was noticeable that poor performer genotypes, RILs 6 and 47 and Gifu, showed consistently lower greenness for both basal and apical leaves, without achieving the same full recovery that was reached by the other genotypes (Figure 7c,d).

### 2.4. Correlations among Traits Aiding Plant Recovery following Submergence 

We explored potential correlations among morphophysiological traits associated with RGR of submerged plants as indicative of their recovery ability (Table 1). S:R was negatively correlated to leaf relative water content (−0.78 < r > −0.69) and stomatal conductance (r = −0.62) during the first week of recovery, meaning that genotypes with high S:R showed poor leaf water status and less open stomata. Moreover, leaf relative water content was positively associated to stomatal conductance (0.73 > r < 0.94), Fv/Fm (0.80 > r < 0.85), and greenness of both basal (0.60 > r < 0.86) and apical leaves (0.68 > r < 0.92). Stomatal conductance correlated in a positive way with Fv/Fm (0.93 > r < 0.95) and leaf greenness (0.79 > r < 0.95). Fv/Fm was positively related to greenness (0.86 > r < 0.95) at all leaf positions (basal and apical). The greenness of basal leaves was positively correlated with that of apical leaves (0.75 > r < 0.91), meaning that that retention of more chlorophyll upon de-submergence occurred both in young and adult leaves. 

## 3. Discussion

This research makes five major contributions to the current knowledge of plant recovery from complete submergence by proving the following: First, the existence of variation in the ability to recover from complete submergence in genotypes of *Lotus japonicus* selected for having different shoot to root dry mass ratio [23]. Second, an evident trade-off between growing during the submergence period and the ability to recover indicated by the negative correlation of the RGRs for each period (submergence vs. recovery) across genotypes. Third, the relationship between RGR and shoot to root ratio (S:R) upon de-submergence, where genotypes with low S:R show better plant performance during early days of recovery. Fourth, recovery of plant water relations assessed by leaf relative water content and stomatal conductance appears essential for early recovery and is correlated negatively with S:R, therefore, S:R can be considered as a rough estimator of the water balance between transpiration (shoot) and water uptake (root) in this model legume. Fifth, the importance of leaf chlorophyll retention in supporting a fast recovery from submergence and suggesting the positive correlation between RGR and greenness of basal and apical plant leaves. Additionally, we reported full repair of the photosynthetic apparatus (i.e., all Fv/Fm with values > 0.8) a few days into the second recovery week. In the following paragraphs, we discuss these eco-physiological traits that aid plants in recovery from complete submergence in *L. japonicus*.

Genotypes showing higher RGRs during submergence later presented lower RGRs during the recovery, and the opposite occurred with the genotypes showing low RGRs when underwater (or even negative values due to tissue loss), as these presented the highest RGRs in the post-submergence phase. This trade-off between growing during submergence versus a sit-and-wait response until water recedes is likely a part of the growth strategies to cope with complete submergence already known as “escape” and” quiescence”, respectively [3,4]. However, this is one of the first reports demonstrating this trade-off across genotypes for a legume species such as *L. japonicus*, as most studies are available for rice ([28,29], but see [30] for pea genotypes). In this regard, it is possible to speculate that genotypes growing during submergence aim at emerging their leaves above the water using carbon reserves (i.e., starch). In contrast, the ones that do not grow when underwater (see [31] for *L. tenuis* cv. Pampa INTA), use their carbon and nutrient reserves more conservatively (i.e., sustaining basal metabolism) until the stress ends and then resume their growth more vigorously, fueled by stored reserves. Further experiments are needed to quantify the level of reserves in *L. japonicus* materials and its relationships with the eco-physiological variables correlating with plant recovery from complete submergence as identified in this work (S:R, leaf relative water content, stomatal conductance, leaf greenness, Fv/Fm as an indicator of photoinhibition) to corroborate this idea.

An adequate plant water status is critical to resume growth after stress either by lack or excess of water (drought or flooding). So, the balance between transpiration and water uptake is essential to sustain high relative water content in tissues and, therefore, enable fast growth [32,33]. In this regard, the shoot to root ratio can have an important role as a trait by impacting plant water balance. It is alleged that shoot growth and increased shoot to root ratio (S:R) during submergence can induce a better balance between gas transport capacity (oxygen source) and root oxygen consumption (oxygen sink) when plants are able to succeed in the process of emerging leaves above water (”escape strategy”; [34,35]). However, an increased S:R can also constrain plant recovery after de-submergence if water loss by transpiration cannot be replenished by root water uptake, regardless of whether leaf emergence was achieved, as seen in the forage grass *Chloris gayana* subjected to 1 week of full submergence [24]. In our experiment, S:R ratio upon de-submergence was negatively related to the RGR during the recovery phase across *L. japonicus* genotypes. Moreover, we found a negative correlation between S:R and leaf relative water content (RWC) that gives support to the idea that the S:R might have impacted on growth resumption due to an impaired water status of plants. Therefore, any environmental condition experienced by plants in their natural environment that can affect their S:R prior to submergence, such as N, P, or K availability [36,37], can impact the subsequent recovery from complete submergence.

Carbon assimilation and growth after de-submergence are influenced by the degree of stomata aperture enabling diffusion of carbon dioxide towards chloroplasts, the N status of leaves, and the functional degree of the photosynthetic apparatus (if damaged) [13,26,38]. In this study, we registered differences among genotypes in stomatal conductance (g_s_), where genotypes with high S:R and low leaf RWC showed lower values of g_s_ until showing recovery for this parameter during the second week after de-submergence. The g_s_ was positively related to plant RGRs across genotypes, particularly during the early recovery. Subsequently, all genotypes achieved full recovery during the second week after submergence ceased. On this note, most works dealing with water excess have reported stomatal conductance during/after waterlogging ([19,23,39,40], among others) but rarely after submergence, so opportunities for comparison are minimal (but see [38]). To illustrate, 6-week-old plants of the flood-sensitive grass *Bromus catharticus* were unable to recover the g_s_ in the same way as controls even after 15 days of recovery from 5 days of complete submergence. In contrast, the flood-tolerant *Phalaris aquatica* did not show, for this parameter, any difference with the controls at any time after de-submergence [38]. In general, *L. japonicus* showed an intermediate behavior compared to the grasses mentioned above, as g_s_ was reduced by complete submergence during the first week, but it was fully recovered in all genotypes during the second week. 

Chlorophyll retention during submergence was positively linked to RGR upon de-submergence both in basal and apical leaves as indicated by the registered leaf greenness dynamics. In this respect, the more tolerant genotypes of *L. japonicus* (RILs 18, 30, and 189) presented lower amounts of (or high sensitivity to) reactive oxygen species (ROS) upon re-aeration (i.e., less oxidative stress), low ethylene formation, and diminished leaf dehydration during recovery compared to the more susceptible ones (RILs 6, 47 and Gifu), similar to what was reported in contrasting accessions of *Arabidopsis thaliana* for submergence tolerance (accessions Lp2-6 vs. Bay-0; [13]). Interestingly, all *L. japonicus* genotypes showed similarly low values for Fv/Fm in young fully expanded leaves, indicating damage to the Photosystem II (PSII) two days after de-submergence. It is known that the re-aeration of leaves (i.e., normoxia) after 7 days in a submergence-induced hypoxic environment can trigger the sudden formation of ROS [41], which ultimately provokes damage to PSII. One week after the stress was released, the genotypes showed a differential ability to recover the Fv/Fm values, and those showing lower values for greenness, g_s_, and leaf RWC (RILs 6, 47 and Gifu) were also the poorest performers. Such differential ability to repair a damaged PSII among genotypes could be related to a differential capacity to mitigate effects of ROS (enzymatically and nonenzymatically), or because genotypes generate different amounts of ROS, or a combination of both. Importantly, all genotypes showed full recovery of Fv/Fm (all values> 0.8) at eleven days after submergence, denoting that damage provoked by submergence was not irreversible and that differences among genotypes are related to the time required for recovery. In contrast to our results for *L. japonicus*, the wetland species *Alternantera philoxeroides* and *Hermarthria altissima* are able to recover functionality of the photosynthetic apparatus after de-submergence in just two days as a result of a rapid acclimation to changing oxygen and/or light conditions [26]. It would be interesting to explore further the ability for photosynthetic acclimation that we found in *L. japonicus* and its variability among genotypes as it is an important trait for adaptation to habitats in which the water level often fluctuates.

On top of the eco-physiological traits contributing to plant recovery from submergence reported here, we are now conducting experiments with new genetic resources for *L. japonicus* to specifically address the role of carbon reserves in recovery ability (e.g., mutants of synthesis or degradation of starch; [42]) as well as a quantitative approach of the ROS signaling, scavenging, and homeostasis to provide a comprehensive network of the factors that mediate post-submergence recovery for this model legume. We think that a better understanding of traits and mechanisms contributing to submergence recovery in *L. japonicus* will undoubtedly help to speed up breeding processes of closely related forage *Lotus* spp. used in current agriculture.

## 4. Materials and Methods 

### 4.1. Species Description

*Lotus japonicus* L. is a small prostrate perennial legume that flowers profusely, being self-fertile. Although it grows slowly during the first weeks after germination, the adult plant has a bushy growth with many branches up to 30 cm in length, which offers abundant material for biochemical and physiological studies [15]. It has a relatively short generation time of three to four months when grown under glasshouse conditions. *L. japonicus* is taxonomically closely related to *L. corniculatus* (birdsfoot trefoil), *L. uliginosus* (big trefoil), and *L. tenuis* (narrow leaf birdsfoot trefoil), which are used in agriculture as pasture legumes [43]. Importantly, this close relationship among forage *Lotus* species and the model legume *L. japonicus* suggests that identification of tolerance traits to abiotic stresses in the model species, such as submergence in this contribution, will be useful to assist and speed up breeding programs and improvement of plant genotypes that are better adapted to environmentally constrained environments prone to flooding.

### 4.2. Plant Material and Growing Conditions

A total of 12 genotypes of *L. japonicus* were used in this study: Gifu B-129 (Gifu) and Miyakojima MG-20 (Miyakojima) and 10 RILs that were derived from a cross between these two parent lines and self-pollination to the F_8_ generation [44]. The RILs were selected from [23] based on having different shoot to root dry mass ratios as we aimed at investigating the role of this trait (related to the potential transpiration/water uptake ratio) on plant growth when recovering from one week of submergence. The seeds were obtained from the National BioResource Project, Miyazaki University (Miyazaki, Japan).

Seeds of each genotype were scarified by rubbing with a fine sand-paper, surface-sterilized with 0.05% (w/v) sodium hypochlorite, rinsed thoroughly in deionized water, and dark-germinated in an incubator (20 °C) in polystyrene boxes containing absorbent water-saturated white paper. After three days, germinated seeds were transplanted to 2 L plastic pots (2 or 3 seedlings per pot) filled with sand and soil with 3.3% of organic carbon (1:1 v/v). After seeding, pots were transferred to a glasshouse at the Faculty of Agronomy at the University of Buenos Aires and during the first week, the seedlings were thinned to one per pot. In order to avoid nutrient limitation for plant growth, 1.2 g of fertilizer (Nitrofull EmergerR, Argentina: 12% N, 5% P, 15% K, 2% Mg, 8% S, 3% Ca, 0.02% Zn, 0.2% Fe, 0.02% Mn, and 0.015% Bo; % are by weight) was added to every pot. Plants were watered daily before treatment imposition.

### 4.3. Experimental Design

After a growth period of 45 days, pots were randomized into two groups and subjected to two treatments for one week: (i) control: pots were watered daily and allowed to drain freely, (ii) complete submergence: plants were submerged in clear water at a depth of 45 cm, which corresponded to a water column of *ca.* 15–20 cm above the top of the plants (water column of 1.5–1.8 times the plant height). Submergence water contained 0.50 mM CaSO_4_, 0.25 mM MgSO_4_, and 1 mM KHCO_3_ (at pH *ca.* 7.5) as in [45]. Dissolved oxygen in the submergence water at midday ranged from 3.9 to 4.6 mg L^–1^ (vs. 7.8 mg L^–1^ in air), measured with a DO-5510 dissolved oxygen meter (LT Lutron Electronic Enterprise, Taipei). The photosynthetic photon flux density (PPFD) reaching control plants was 1100 ± 180 µmol m^−2^ s^−1^, while for submerged plants, it was 490 ± 80 µmol m^−2^ s^−1^ (LI-192 Underwater Quantum Sensor; Li-Cor Inc., Lincoln, NE, USA). The latter is a light environment that allows for underwater photosynthesis in C_3_ species [46]. During the first week of the experiment, all plants (both assigned to control and submergence treatments) were placed in plastic containers (0.8 m × 0.5 m × 0.5 m) to facilitate the submergence imposition. After the first experimental week, plants were taken out of the containers and rotated weekly within the glasshouse during a two-week recovery period. The number of replicates per genotype and treatment combination varied from 6 to 8 according to the number of available plants. Average glasshouse relative humidity was 62% ± 12%, and the average air temperature was 24/16 °C (day/night). 

### 4.4. Dry Mass and Relative Growth Rate

Plants of all genotypes were harvested at the beginning of the experiment (day 0), at day 7 (end of the submergence), at day 14 (end of the first recovery week, to infer ‘early recovery ability’ from submergence), and at day 21 (end of the second recovery week, to infer ‘late recovery ability’). The dry mass of plants was obtained after drying at 80 °C for 72 h (i.e. until constant weight). In the second harvest (immediately after submergence; day 7), shoots were separated from roots to calculate the shoot to root dry mass ratio of the different genotypes upon de-submergence.

Relative growth rate of plants (RGR) after submergence was used as an indicator of recovery ability of the genotypes. In this study, the identity of each genotype per se was not particularly relevant as to have sufficient genetic variability for some traits, such as shoot to root dry mass ratio, to potentially influence plant water balance upon de-submergence and return to oxygenated well-drained conditions. The RGR was calculated following the equation by [47]:

RGR (g g^−1^ d^−1^) = [ln(W_2_) − ln(W_1_)] / (t_2_ − t_1_) (1)

where W_2_ and W_1_ are the plant dry weights from the corresponding treatment at times 2 and 1, respectively, with the value of W_1_ being the treatment average, and t_2_ − t_1_ is the number of days between sampling times (i.e., 7 d).

### 4.5. Leaf Physiological Responses: Relative Water Content, Stomatal Conductance, Chlorophyll Fluorescence, and Greenness 

Leaf physiological measurements were made at days 2 and 7 during the first recovery week (days 9 and 14 since the beginning of the experiment), and at days 10 and 14 following de-submergence during the second recovery week (days 17 and 21 of the experiment). These measurements aimed at monitoring the recovery of leaf water status, stomatal functioning, and the impact of one week of full submergence on leaf greenness as indicators of nitrogen status of basal and apical leaves due to damage to the photosynthetic apparatus through measurements of chlorophyll fluorescence of dark-adapted young leaves (i.e., Fv/Fm). For the last parameter, measurements were performed at days 2 and 7 during the first recovery week and at day 11 during the second recovery week. The number of replicates taken for the physiological measurements was five per genotype and treatment combination.

Relative water content of leaves (RWC) was measured according to the methodology proposed by [48]. First, young, fully expanded leaves located at a top position of plants were taken and immediately weighed to obtain their fresh weight. Second, samples were incubated for 12 h in tightly closed tubes containing deionized water to get their turgid weight. Third, the leaves were oven-dried at 70 °C for 48 h to obtain their dry weight (the weights of leaves were latter added to the corresponding plants). Finally, the RWC of leaves was obtained as: [(fresh weight – dry weight) / (turgid weight – dry weight)] × 100.

Stomatal conductance of young, fully expanded leaves at top positions was measured near midday by using a leaf porometer (model SC-1; Decagon Devices, Pullman, WA, USA) to evaluate water loss by transpiration and facilitation of CO_2_ diffusion into leaves for photosynthesis. Leaf greenness was measured in young (apical) and old (basal), fully expanded leaves by using a portable chlorophyll meter (SPAD-502; Konica Minolta Sensing, Osaka, Japan). This parameter infers that leaf yellowing is indicative of chlorophyll degradation, nitrogen remobilization, and anticipated leaf senescence as consequences of the impact of submergence stress. Chlorophyll fluorescence (Fv/Fm) was measured on top-most, fully expanded leaves after a dark-adaptation period of 20 min by using leaf clips and the OS-30p portable fluorometer (Opti-Sciences Inc., USA). This parameter indicates the proportion of functional Photosystem II (PSII) reaction centers so that it can be used to quantify the degree of photoinhibition by damage to the PSII [49].

### 4.6. Statistical Analyses 

RGR and shoot to root dry mass ratio responses during submergence were evaluated by two-way ANOVAs, with ’submergence” and ”genotype“ as main factors. When significant interactions were detected, the least significant difference (LSD) and Fisher’s protected tests were used to determine the effect of treatments among genotypes. During the recovery phase, regression analyses were performed to explore relationships between plant RGR and shoot to root dry mass ratio, leaf relative water content, stomatal conductance, chlorophyll fluorescence of dark-adapted leaves (Fv/Fm), and greenness of basal (adult) and apical (young) leaves. These relationships were examined separately for the first and the second recovery weeks to identify traits associated with ‘early’ and ‘late’ recovery responses. Additionally, relations between physiological variables that were significantly related to RGR during recovery were explored through Pearson correlations. Normality and the homogeneity of variances were verified before each analysis. All results are presented as means ± standard errors. Statistical analyses were done using Infostat software [50], and graphs were made with GraphPad Prism 5 for Windows (GraphPad Software, San Diego, CA, USA). 

## 5. Conclusions

We found variation in the recovery ability from short-term complete submergence in genotypes of the model legume *Lotus japonicus*. The ability to recover assessed through plant RGR post-submergence negatively correlated with plant RGR displayed during the submergence period, which indicates the existence of a trade-off between growing during vs. after the stress. Several eco-physiological traits and responses correlating to recovery ability across genotypes were identified, which were particularly important in explaining the recovery of plants in the first week after de-submergence. Shoot to root dry mass ratio (S:R) was inversely related to RGR during the recovery. Leaf relative water content and stomatal conductance were positively related to RGR in the early recovery week, and negatively related to S:R ratio, which is why the S:R ratio can be used as an estimator of the potential plant water balance (transpiration in shoot vs. water uptake in root) in post-submergence. Leaf greenness of basal and apical leaves was positively linked to RGR during early days after submergence, indicating that chlorophyll retention is important for a fast recovery. The full repair of the submergence-damaged photosynthetic apparatus occurred later than the recovery of plant water status, and it was completed during the second recovery week. The inclusion of traits contributing to submergence recovery in *L. japonicus* as identified in this work should be considered to speed up breeding processes of closely related forage *Lotus* spp. used in current agriculture.

## Figures and Tables

**Table 1:** Pearson correlation coefficients for the morphophysiological traits measured over the recovery phase on 12 genotypes of *Lotus japonicus* (10 recombinant inbred lines and their parents Gifu and MG20) subjected to control (above the diagonal) for three weeks or complete submergence conditions for one week (below the diagonal) and allowed to grow for another 2 weeks. Only correlations for morphophysiological traits that were significantly related to RGR during the recovery from submergence are shown (data from Figure 3, Figure 4, Figure 5, Figure 6 and Figure 7). Abbreviations are S:R (shoot to root dry mass ratio at de-submergence), LRWC (leaf relative water content), g_s_ (stomatal conductance), Fv/Fm (chlorophyll fluorescence of dark-adapted leaves). Significant differences: *, *p* < 0.05; **, *p* < 0.001; ns, *p* > 0.05.

Controls ► Recovering from sub. ▼	S:R	LRWC 2-d Recovery	LRWC 7-d Recovery	g_s_ 2-d Recovery	g_s_ 7-d Recovery	Fv/Fm 7-d Recovery	Greenness Basal Leaves 2-d Recovery	Greenness Apical Leaves 2-d Recovery	Greenness Basal Leaves 7-d Recovery	Greenness Apical Leaves 7-d Recovery
S:R		0.15 ^ns^	0.22 ^ns^	0.18 ^ns^	−0.02 ^ns^	0.50 ^ns^	−0.04 ^ns^	−0.18 ^ns^	−0.21 ^ns^	0.27 ^ns^
LRWC 2-d recovery	−0.69 *		0.31 ^ns^	0.22 ^ns^	0.16 ^ns^	−0.22 ^ns^	0.38 ^ns^	−0.17 ^ns^	−0.20 ^ns^	−0.14 ^ns^
LRWC 7-d recovery	−0.78 **	0.94 **		0.07 ^ns^	0.26 ^ns^	−0.29 ^ns^	0.13 ^ns^	0.33 ^ns^	−0.26 ^ns^	0.25 ^ns^
g_s_ 2-d recovery	−0.62 *	0.91 **	0.85 **		0.54 ^ns^	0.25 ^ns^	0.31 ^ns^	−0.21 ^ns^	−0.28 ^ns^	0.58 *
g_s_ 7-d recovery	−0.51 ^ns^	0.75 *	0.73 *	0.85 **		−0.15 ^ns^	0.45 ^ns^	0.30 ^ns^	0.04 ^ns^	0.43 ^ns^
Fv/Fm 7-d recovery	−0.54 ^ns^	0.85 **	0.80 **	0.93 **	0.95 **		−0.03 ^ns^	−0.09 ^ns^	−0.24 ^ns^	0.31 ^ns^
Greenness basal leaves 2-d recovery	−0.51 ^ns^	0.86 **	0.76 *	0.95 **	0.88 **	0.92 **		−0.52 ^ns^	0.22 ^ns^	−0.08 ^ns^
Greenness apical leaves 2-d recovery	−0.44 ^ns^	0.68 *	0.60 *	0.83 **	0.92 **	0.93 **	0.87 **		0.40 ^ns^	−0.72 *
Greenness basal leaves 7-d recovery	−0.58 *	0.92 **	0.85 **	0.88 **	0.79 *	0.86 **	0.87 **	0.75 *		0.26 ^ns^
Greenness apical leaves 7-d recovery	−0.46 ^ns^	0.80 **	0.68 *	0.90 **	0.88 **	0.95 **	0.91 **	0.94 **	0.84 **	

## Supplementary Materials

The following are available online at https://www.mdpi.com/2223-7747/9/4/538/s1, **Table S1**: Plant dry mass (g per plant) of 12 genotypes of *Lotus japonicus* (10 recombinant inbred lines and their parents Gifu and MG20) harvested throughout the experiment. Values are means ± standard errors of 6–8 replicates. **Table S2**: Leaf relative water content (RWC; %) on the top-most, fully expanded leaves of control plants of *Lotus japonicus* recombinant inbred lines (RIL) and the parental MG20 and Gifu lines. Values are means ± standard errors of 5 replicates. For further details, see Materials and Methods Section. **Table S3**: Stomatal conductance, g_s_ (mmol m^−2^ s^−1^), of the top-most, fully expanded leaves of control plants of *Lotus japonicus* recombinant inbred lines (RIL) and the parental MG20 and Gifu lines. Values are means ± standard errors of 5 replicates. For further details, see Materials and Methods Section. **Table S4**: Dark-adapted chlorophyll fluorescence (Fv/Fm) on the top-most fully expanded leaves of control plants of *Lotus japonicus* recombinant inbred lines (RIL) and the parental MG20 and Gifu lines. Values are means ± standard errors of 5 replicates. For further details, see Materials and Methods Section. **Table S5**: Greenness (SPAD units) of basal and apical, young, fully expanded leaves of control plants of *Lotus japonicus* recombinant inbred lines (RIL) and their parental MG20 and Gifu lines. Values are means ± standard errors of 5 replicates. For further details, see Materials and Methods Section.
