# Supplementary material for: Eco-Physiological Traits Related to Recovery from Complete Submergence in the Model Legume Lotus japonicus
Source: Plants (Basel). 2020 Apr 21;9(4):538. doi: 10.3390/plants9040538 (PMC7238009; doi:10.3390/plants9040538)
Supplement: Supplementary file 1 [file plants-09-00538-s001.pdf]

plants

MDPI

Eco-physiological traits related to recovery from complete submergence in the model legume *Lotus japonicus*

F.B. Buraschi, F.P.O. Mollard, A.A. Grimoldi and G.G. Striker

SUPPLEMENTARY MATERIAL

Table S1. Plant dry mass (g per plant) of 12 genotypes of *Lotus japonicus* (10 recombinant inbred lines and their parents Gifu and MG20) harvested along the experiment. Values are means ± standard errors of 6-8 replicates.

Genotype	Initial	After one-week submergence		After one-week recovery		After two-week recovery	
	Control	Control	Submerged	Control	Submerged	Control	Submerged
RIL-6	0.45±0.08	0.68±0.07	0.59±0.05	0.83±0.12	0.76±0.08	1.80±0.11	0.94±0.20
RIL-18	0.51±0.05	0.63±0.04	0.28±0.05	1.40±0.12	0.64±0.17	1.60±0.13	0.97±0.18
RIL-30	0.33±0.06	0.57±0.13	0.24±0.07	0.74±0.23	0.60±0.09	0.91±0.05	0.80±0.17
RIL-47	0.28±0.05	0.66±0.13	0.58±0.14	1.03±0.20	0.70±0.05	1.22±0.19	0.86±0.37
RIL-80	0.28±0.02	0.52±0.08	0.18±0.03	0.78±0.33	0.38±0.08	1.37±0.10	0.91±0.07
RIL-82	0.46±0.09	0.76±0.11	0.31±0.01	1.04±0.32	0.61±0.17	1.90±0.32	0.98±0.15
RIL-85	0.41±0.02	0.53±0.04	0.25±0.03	0.77±0.11	0.47±0.06	0.99±0.15	0.69±0.05
RIL-176	0.22±0.06	0.31±0.03	0.14±0.02	0.84±0.22	0.31±0.09	2.29±0.45	0.84±0.17
RIL-189	0.33±0.07	0.45±0.06	0.21±0.05	0.94±0.22	0.50±0.10	1.75±0.46	1.24±0.17
RIL-200	0.25±0.06	0.36±0.06	0.13±0.02	0.94±0.12	0.28±0.03	1.28±0.11	0.66±0.10
Gifu	0.14±0.02	0.30±0.05	0.15±0.02	0.58±0.04	0.23±0.04	0.93±0.17	0.46±0.08
MG20	0.54±0.10	0.80±0.15	0.45±0.08	1.46±0.10	0.90±0.33	2.39±0.34	1.01±0.20

Table S2. Leaf relative water content (RWC; %) on the top-most fully expanded leaves of control plants of *Lotus japonicus* recombinant inbred lines (RIL) and the parental MG20 and Gifu. Values are means ± standard errors of 5 replicates. For further details see Materials and methods section.

Genotype	Values for control plants			
	LRWC (%) after 2-d of recovery	LRWC (%) after 7-d of recovery	LRWC (%) after 10-d of recovery	LRWC (%) after 14-d of recovery
RIL-6	89.2±1.4	91.4±1.0	91.0±1.1	90.8±0.9
RIL-18	90.8±0.8	90.4±1.1	91.8±1.1	90.4±0.5
RIL-30	91.2±1.0	91.0±1.1	90.4±1.8	91.0±1.0
RIL-47	92.0±1.2	91.2±1.0	91.0±1.0	90.4±0.9
RIL-80	91.2±0.7	91.6±1.0	91.4±0.9	90.2±0.9
RIL-82	91.4±0.9	91.1±1.0	91.8±1.1	90.8±1.0
RIL-85	92.4±1.3	91.6±0.9	91.0±1.0	91.8±0.7
RIL-176	91.2±1.0	90.2±1.5	91.2±1.1	91.8±0.9
RIL-189	91.2±1.0	90.2±1.5	91.2±1.1	91.8±0.9
RIL-200	90.4±1.1	90.0±0.6	90.0±0.7	90.6±1.0
Gifu	91.6±1.4	92.2±1.0	90.2±1.0	91.2±0.7
MG20	91.6±1.4	92.0±0.7	91.4±0.8	91.4±1.3
ANOVA for the effect of genotype	p-value: 0.86	p-value: 0.91	p-value: 0.98	p-value: 0.94

Table S3. Stomatal conductance-g_s (mmol m^{-2} s^{-1}) on the top-most fully expanded leaves of control plants of *Lotus japonicus* recombinant inbred lines (RIL) and the parental MG20 and Gifu. Values are means ± standard errors of 5 replicates. For further details see Materials and methods section.

Genotype	Values for control plants			
	g_s after 2-d of recovery	g_s after 7-d of recovery	g_s after 10-d of recovery	g_s after 14-d of recovery
RIL-6	194.2±9.4	191.4±14.0	198.8±12.5	200.4±8.1
RIL-18	189.0±7.9	187.8±6.5	194.4±13.3	200.8±6.9
RIL-30	189.4±5.6	199.0±10.3	187.2±6.4	194.6±10.1
RIL-47	201.2±9.9	191.4±5.1	196.8±9.7	201.0±10.2
RIL-80	197.6±5.5	198.0±11.8	198.8±12.3	202.2±8.4
RIL-82	199.2±13.2	197.4±7.3	203.2±8.4	199.0±8.3
RIL-85	194.0±8.4	192.6±8.9	196.8±7.4	204.8±11.8
RIL-176	197.4±4.8	193.2±7.8	200.0±13.6	208.8±9.2
RIL-189	204.6±9.8	199.0±13.0	203.2±8.4	200.2±9.4
RIL-200	200.8±9.2	198.8±11.0	193.2±8.5	198.2±4.7
Gifu	203.2±8.1	202.0±8.0	199.0±13.7	193.4±7.7
MG20	199.8±9.8	199.0±8.9	204.2±12.8	205.8±7.0
ANOVA for the effect of genotype	*p*-value: 0.82	*p*-value: 0.97	*p*-value: 0.93	*p*-value: 0.96

Table S4. Dark-adapted chlorophyll fluorescence (Fv/Fm) on the top-most fully expanded leaves of control plants of *Lotus japonicus* recombinant inbred lines (RIL) and the parental MG20 and Gifu. Values are means ± standard errors of 5 replicates. For further details see Materials and methods section.

Genotype	Values for control plants		
	Fv/Fm after 2-d of recovery	Fv/Fm after 7-d of recovery	Fv/Fm after 11-d of recovery
RIL-6	0.810±0.004	0.808±0.006	0.807±0.004
RIL-18	0.810±0.007	0.808±0.006	0.804±0.009
RIL-30	0.816±0.006	0.803±0.005	0.814±0.009
RIL-47	0.807±0.006	0.806±0.006	0.814±0.005
RIL-80	0.810±0.009	0.805±0.005	0.811±0.004
RIL-82	0.810±0.009	0.805±0.005	0.810±0.004
RIL-85	0.810±0.004	0.807±0.007	0.813±0.007
RIL-176	0.808±0.004	0.810±0.005	0.808±0.004
RIL-189	0.812±0.009	0.808±0.008	0.809±0.004
RIL-200	0.808±0.004	0.810±0.005	0.808±0.004
Gifu	0.807±0.007	0.806±0.006	0.806±0.010
MG20	0.807±0.006	0.811±0.003	0.810±0.007
ANOVA for the effect of genotype	*p*-value: 0.93	*p*-value: 0.94	*p*-value: 0.96

Table S5. Greenness (SPAD units) of basal and apical young fully expanded leaves of control plants of *Lotus japonicus* recombinant inbred lines (RIL) and their parental MG20 and Gifu. Values are means ± standard errors of 5 replicates. For further details see Materials and methods section.

	Values for controls – Basal leaves			
Genotype	Greenness 2-d of recovery	Greenness 7-d of recovery	Greenness 10-d of recovery	Greenness 14-d of recovery
RIL-6	33.6±1.3	35.4±1.4	35.8±1.7	34.2±1.5
RIL-18	35.6±2.1	36.4±2.2	35.2±2.0	34.2±1.5
RIL-30	35.2±2.7	37.0±1.6	35.8±2.2	36.4±2.1
RIL-47	35.2±2.9	36.4±2.1	36.8±2.0	34.8±2.2
RIL-80	34.8±2.4	36.2±2.2	36.2±1.5	36.0±1.4
RIL-82	35.2±1.8	35.6±2.7	36.0±2.2	35.2±2.0
RIL-85	34.2±2.7	35.0±2.4	35.8±2.3	36.4±1.8
RIL-176	35.0±1.6	35.2±2.4	34.8±2.1	35.2±2.3
RIL-189	35.0±2.1	34.8±2.4	36.6±1.7	36.2±1.8
RIL-200	35.2±2.1	37.4±2.1	37.6±1.3	35.2±2.1
Gifu	36.6±2.0	35.6±2.0	35.0±2.4	36.4±2.4
MG20	35.6±1.7	35.4±2.0	37.6±1.4	36.0±2.4
ANOVA for the effect of genotype	*p*-value: 0.89	*p*-value:0.90	*p*-value:0.88	*p*-value:0.87

	Values for controls – Greenness of apical fully-expanded leaves			
	Greenness 2-d of recovery	Greenness 7-d of recovery	Greenness 10-d of recovery	Greenness 14-d of recovery
RIL-6	43.2±0.6	43.0±1.0	42.0±1.3	42.6±1.1
RIL-18	41.8±0.9	41.6±0.8	41.0±0.7	41.8±1.0
RIL-30	42.0±1.0	41.8±1.4	41.6±1.2	41.8±1.2
RIL-47	42.0±1.0	41.8±1.2	42.6±1.0	41.4±1.4
RIL-80	41.6±1.2	42.0±0.7	42.0±0.9	42.0±1.1
RIL-82	41.6±0.9	42.6±0.5	42.0±1.0	42.6±0.6
RIL-85	43.0±0.3	42.2±1.1	41.0±0.5	42.6±0.9
RIL-176	41.8±1.1	42.4±1.0	42.2±0.6	42.8±0.7
RIL-189	42.0±0.8	43.4±0.6	42.6±1.1	42.2±1.0
RIL-200	41.6±0.8	42.0±1.2	42.4±0.9	42.0±1.2
Gifu	42.4±1.1	43.0±1.0	42.0±0.9	42.8±1.0
MG20	41.6±0.9	43.0±1.0	43.0±1.1	42.2±1.1
ANOVA for the effect of genotype	*p*-value:0.88	*p*-value:0.89	*p*-value:0.87	*p*-value:0.91

© 2020 by the authors. Submitted for possible open access publication under the terms and conditions of the Creative Commons Attribution (CC BY) license (http://creativecommons.org/licenses/by/4.0/).
